# Self-management experiences among men and women with type 2 diabetes mellitus: a qualitative analysis

**DOI:** 10.1186/1471-2296-13-122

**Published:** 2012-12-19

**Authors:** Rebecca Mathew, Enza Gucciardi, Margaret De Melo, Paula Barata

**Affiliations:** 1 Northern Ontario School of Medicine, East Campus, Laurentian University, 935 Ramsey Lake Road, Sudbury, Ontario, P3E 2C6, Canada; 2 School of Nutrition, Ryerson University, 350 Victoria Street, Toronto, Ontario, M5B 2K3, Canada; 3 University Health Network Diabetes Education Center, Toronto, Ontario, Canada; 4 Department of Psychology, University of Guelph, 4005 - Mackinnon Extension, Guelph, Ontario, Canada

**Keywords:** Diabetes, Self-management, Type 2 Diabetes Mellitus, Sex, Gender

## Abstract

**Background:**

The purpose of this study is to better understand differences in diabetes self-management, specifically needs, barriers and challenges among men and women living with type 2 diabetes mellitus (T2DM).

**Methods:**

35 participants were recruited from a diabetes education center (DEC) in Toronto, Canada. Five focus groups and nine individual interviews were conducted to explore men and women's diabetes self-management experiences.

**Results:**

The average age of participants was 57 years and just over half (51.4%) were female. Analyses revealed five themes: disclosure and identity as a person living with diabetes; self-monitoring of blood glucose (SMBG); diet struggles across varying contexts; utilization of diabetes resources; and social support. Women disclosed their diabetes more readily and integrated management into their daily lives, whereas men were more reluctant to tell friends and family about their diabetes and were less observant of self-management practices in social settings. Men focused on practical aspects of SMBG and experimented with various aspects of management to reduce reliance on medications whereas women focused on affective components of SMBG. Women restricted foods from their diets perceived as prohibited whereas many men moderated their intake of perceived unhealthy foods, except in social situations. Women used socially interactive resources, like education classes and support groups whereas men relied more on self-directed learning but also described wanting more guidance to help navigate the healthcare system. Finally, men and women reported wanting physician support for both affective and practical aspects of self-management.

**Conclusions:**

Our findings highlight the differences in needs and challenges of diabetes self-management among men and women, which may inform gender-sensitive diabetes, care, counseling and support.

## Background

In 2008, an estimated 1.6 million Canadians had clinically diagnosed diabetes mellitus (DM) [1], representing 5.5% of all women and 6.2% of all men [2]. Diabetes can lead to cardiovascular mortality [3,4] and microvascular complications, such as nephropathy [5], neuropathy [6,7], and retinopathy [8]. For instance, Canadians with T2DM were approximately 3 times more likely to be hospitalized with a myocardial infarction or stroke than their counterparts without diabetes [9] and are twice as likely to die prematurely compared to those without diabetes [10]. The literature also supports sex differences with respect to morbidity and mortality: women with T2DM have a greater risk of death from cardiovascular disease [11] and stroke [4] compared to men. These differences in mortality may be linked to variations in how men and women experience and manage their diabetes [12].

The relevant body of literature reports differences in diabetes management experiences between men and women, particularly in their beliefs, attitudes, fears and concerns about the disease. Women, more often than men, view diabetes as negatively affecting their lives. At diagnosis, more women reported fearing loss of health, diabetes-related morbidity, and early mortality compared to men [13]. For instance, women worry more about both acute and chronic diabetes complications such as hypoglycemia [14], and chronic complications, like cardiovascular and renal disease [13]. Women also report significantly more depressive symptoms [15], which can lower their participation in diabetes education and medication compliance [16]. Men are more concerned that diabetes will constrain their lifestyles [14] but believe it is controllable [17]. Men report being more concerned about how diabetes affects their provider role [18], whereas women worry more about how self-care will hinder their familial responsibilities [19], and they also tend to sacrifice their dietary regimen for their family’s food preferences [19].

There is also literature demonstrating differences in barriers to self-management of T2DM [20,21] but limited studies examining gender specific differences. For example, Nagelkerk et al. completed a qualitative study in which the most commonly reported barriers among patients with diabetes lack of understanding of the overall plan of care and frustration from inadequate metabolic control and progressive disease despite compliance with the self-care recommendations [22]. A study by Whittemore et al. among women with diabetes demonstrated more positive outcome measures, including metabolic control, diet and diabetes-related distress with increased self-confidence in living with diabetes and positive social supports [23]. This study suggests that barriers to diabetes self-care among women might include lack of self-confidence and inadequate support from their immediate friends and family. Another qualitative study by Cherrington et al. examining barriers and facilitators to diabetes self-care among Latino immigrants found that women’s barriers related to inadequate social support at home, especially related to food preparation. On the other hand, the predominant barriers to self-care faced by men were lack of flexibility and schedule intensity within the work place [24]. Despite some literature describing these gender differences, there is still a gap in comprehensive narrative accounts of the management experiences of men and women living with diabetes. A better understanding of how diabetes self-management experiences are similar or different between men and women can provide insight to better direct strategies to enhance care and management support to those living with diabetes.

Therefore, the objective of our study is to explore diabetes management experiences, specifically needs, challenges, and barriers identified by men and women using qualitative research methods. This methodology will allow us to understand issues and life experiences that are meaningful and significant to those living with diabetes. Our intention is to build upon the body of evidence to better develop a gender-sensitive understanding of diabetes self-management and discover ways to better integrate gender-specific care into current interventions and clinical practice.

## Methods

Our rationale for selecting this study technique was that there are limited studies that have focused on narrative accounts of how men and women experience self-care of their diabetes mellitus using a data-driven approach to their analysis. The ethics approval for this research study was obtained from the University Health Network Research Ethics Board in Ontario, Canada. The initial data collection for this study was completed between 2006–2007.

### Study design

This original research question is broad, so as to accommodate for inductive, data-driven thematic analysis. We chose this qualitative method of study in order to elaborate on the multi-dimensional aspects of diabetes self-management for men and women, and compare and contrast these experiences in order to better understand differences. Of note, this study was initially undertaken to examine differences in diabetes self-care among users and non-users of the Diabetes Education Center. While our study results are drawn from a secondary analysis we feel as though they can provide meaningful contribution to the current body of literature.

We utilized a self-management framework by Brewer-Lowry et al [25]. to direct our interview guide. This framework describes diabetes self-management as a series of intersections between various tasks (diet, physical activity, glucose monitoring at home, foot care, medications, and seeking medical care) and resources (self-care, informal, formal and medical care). This model is a clear example of the multi-faceted nature of diabetes self-management: struggles within a single domain can have substantial effects on both immediate and long-term outcomes in multiple areas. Brewer-Lowry et al. also discuss the various types of resources that patients with T2DM utilize in order to practice their self-care behaviors, such as family, friends, formal support and medical care. While this model was useful in directing our interview guide, our major themes were developed based on our inductive analysis and the emerging self-management issues identified by our study population.

Please see Appendix 1 for the detailed interview guide. Focus groups began with introductions and general discussions about life circumstances such as when participants were diagnosed, how long have they lived with diabetes and their motivation for participating in the focus group. The rest of the focus group was structured around a prepared list of open-ended questions regarding diabetes self-management issues that was developed into a topic guide to elicit discussion. The topic guide for the individual interviews was exactly the same as that for the focus groups. All focus groups and interviews were audio-recorded and subsequently transcribed into Nvivo 2.0. The interview transcripts were kept in password protected and secure hard drives at our research lab throughout all stages of data analysis to ensure confidentiality and data security. Following initial data collection, all identifying information of the participants was removed and objective identifiers were used on the transcripts to ensure anonymity.

### Participant recruitment

A research assistant identified eligible patients from the DEC’s clinical charts and, using a standardized script, telephoned patients to inform them of the study. Because we had difficulty arranging a common time for all who were interested in participating in the focus groups, we offered those who were not able to attend the option to participate by phone in an individual interview. To those who agreed to participate in a focus group, a letter with details and a map was mailed and a reminder phone call was made the day before the focus group. For those who attended the focus group, consent forms were reviewed and signed, and for individuals participating in telephone interviews, consent was provided over the phone. A $25 honorarium was offered to cover travel and parking costs at the DEC. Of the 35 participants, nine engaged in telephone interviews and the remaining participants were engaged in focus groups of three to eight participants each based on participant availability.

### Study population

The sampling technique required every DEC participant who met inclusion criteria to be notified of the study and this database was used to ensure representative sampling of the population in a at the DEC, situated in a large, urban, culturally diverse city in Ontario, Canada. All participants had T2DM; first visited the DEC between October 2002 and December 2003; spoke, read, and wrote English; and were self-managing their diabetes. We excluded patients who were pregnant, and/or on dialysis. We conducted five focus group: one mixed sex group, two with only men and two with only women to allow for sex specific discussions. The focus groups were distributed in this manner to allow for sex-specific discussions of personal issues among participants that may not have surfaced if all groups were mixed-sex. Interviews lasted 1–2 hours and were held in one of the Diabetes Education Centers from which the participants had been initially recruited.

### Data analysis

The research team addressed issues of rigor and trustworthiness based on a quality framework outlined by Meyrick [26]. Our research team was composed of one academic expert in diabetes (EG), one practice expert (MD), one expert in qualitative research (PB) and a resident physician with a special interest in diabetes (RM). Three of the investigators (PB, MD, EG) performed the focus group and individual interviews but the transcripts had all identifying information removed so we were able to maintain confidentiality and anonymity during analysis. Our data analysis was made inductively as we created our initial themes without having a coding scheme in place.

We used thematic analysis to explore salient topics that emerged from the focus groups and interviews [27]. Our thematic analysis involved initial independent coding by three authors (EG, MD, PB). Seventy-eight codes were identified in the preliminary analysis of the transcripts. Codes were then clustered and used to form 13 preliminary sub-themes that integrated several of the originally identified codes and encompassed more general topics that were the focus of the transcripts. The preliminary sub-themes were then analyzed by sex where similarities and differences among men and women were examined and examples of these themes (i.e. direct quotations) were identified within the transcripts. Subsequently, word documents were created around each sub-theme with relevant quotations selected from the transcripts that highlighted the sub-theme. Further analyses led to the emergence of five overarching themes that illustrated the most significant and broad similarities or differences of diabetes self-management experiences between men and women. The five overarching themes identified were: identity and disclosure as a person living with diabetes, self-monitoring of blood glucose levels, struggles with diet and nutrition, utilization of diabetes resources and social supports. These themes are further elaborated upon in the results section and the relevant quotations used as support for the inclusion and development of the themes.

## Results

Participant demographics are listed in Table 1. There were a total of 35 participants and the average age was 57 years, with 51% female and 49% male participants. Almost half of the study population achieved some or full completion of high school education (49%), had a family yearly income of below $ 29, 999 (43%) and were married or living with a partner (43%). More than half of the participants (63%) were foreign-born from various ethnic backgrounds. Data analysis yielded five emergent themes addressed by both men and women: identity and disclosure as a person living with diabetes, self-monitoring of blood glucose levels, struggles with diet and nutrition across varying contexts, utilization of diabetes resources and social support. Please see Table 2 for a summary of the gender differences divided by overarching theme.

**Table 1:** Demographic characteristics of participants

***Demographic***	***Men (n = 17)***	***Women (n = 18)***	***Total (n = 35)***
**Age (average, in years)**	55	64	57
**Marital Status (number of participants)**			
Single/never married	1	7	8
Married/living with partner	7	8	15
Separated/divorced	7	1	8
Widowed	0	2	2
Unreported	2	0	2
**Education (number of participants)**			
Less than grade 9	1	2	3
Some/completed high school	5	11	17
Some/completed college or university	5	4	10
Graduate/professional degree	3	0	3
Unreported	3	1	4
**Employment (number of participants)**			
Full/part-time, self-employed	9	2	11
Unemployed	2	5	7
Retired	3	9	14
Student	0	1	1
Unreported	3	1	4
**Family Income (number of participants)**			
Below $29, 999	4	11	15
$30, 000 – 79, 000	7	2	10
$80, 000 and above	2	1	3
Unreported	4	4	8
**Canadian-born (number of participants)**			
Yes	4	5	9
No	9	13	22
Unreported	4	0	4
**DEC Status(number of participants)**			
Non-user	8	6	14
User	7	12	19
Unreported	2	0	2

**Table 2:** Summary of similarities and differences between men and women, divided by five overarching themes

	**Identity and disclosure as person living with diabetes**	**Self-monitoring of blood glucose**	**Diet struggles**	**Utilization of diabetes resources**	**Social support**
**Men**	Reluctant to disclose, kept diagnosis secret	Self-experimentation with diet, exercise, insulin, medication	Adherence in social settings, in context of lack of disclosure of diabetes	Self-directed, independent resources	Majority of support from spouse, especially diet changes
**Women**	Open about identity, self-labeled as having diabetes	Affective fears, anxieties about monitoring	Difficulties with restriction, focus on ‘cheating’ on diet	Socially interactive resources, classes and support groups	Mixed sources, family, friends and children
**Both**	Acceptance after adjustment period following diagnosis	Emphasis on body cues that linked to blood glucose levels	Frustration of adherence to dietary recommendations	Made use of available resources, sought out resources	Strong support from physicians, health care professionals

### Identity and disclosure as a person living with diabetes

Women openly identified themselves to others as having diabetes in contrast to men who often hid their diagnosis in social settings and sometimes kept the diagnosis from family and friends. Among the women’s focus groups, sources of public identification, including medical alert bracelets, were discussed and perceived as very useful to have, as it was a clear and easy identifier of their health condition to others. One female participant noted that

"It doesn’t hurt to have that little bracelet that says this is one of my problems…I have it in my wallet and it says I’m a diabetes person, I’m unconscious, and it mentioned it [FG5.F6]."

Women also emphasized how their diagnosis was well known to family and friends, who often helped them stay on track with their self-care recommendations. On the other hand, throughout the male focus groups and interviews, no specific public identifiers of diabetes were mentioned, and some men repeatedly discussed worry and anxiety over the thought of family and friends learning about their diabetes:

"In my family, no one knows that I have diabetes…I don’t want to let it out. I’m too young, and I don’t want to let them know that I’m a diabetic. [I,M8]."

Females’ response to initial diagnosis was less dramatic and substantially less focused on thoughts of imminent mortality; women’s discussions were more rooted in feelings of sadness and disappointment instead of life threatening sequelae when they were first diagnosed. However, expressions of shame, embarrassment, and imminent thoughts of death upon diagnosis with diabetes were more often noted among men; one man reported:

"So when I was diagnosed with diabetes, I thought I was going to die. [I] thought I was different than the others. I couldn’t face people, because I said, ‘Oh, this is a terrible sickness! Oh my god, I’m going to die!’ [FG1,M5]"

Differences in observance of nutrition self-care practices were particularly apparent in how men and women discussed maintaining diet and lifestyle changes in social settings. When asked how they cope with diet in social settings, one woman said,

"If I go out, and if it’s a place I know that has a microwave, I’ll bring my own food…and I’ll just use their microwave. Even if I have dinner at their place, they’ll eat their food, and I’ll eat mine. [I,F7]"

The idea of making adjustments in lifestyle to better observe self-care recommendations was consistent across numerous discussions in the women’s focus groups and interviews. Women seem much more able to publically identify themselves as having diabetes, and in turn, align their public identities with diabetes self-care behaviors. However, men expressed difficulty in reconciling diabetes with social activities. Some even avoided activities that might tempt them to stray from dietary recommendations. One man reported,

"For the past, let’s say 6 months or so, I take it [diabetes self-care] really serious. Places I’m invited to, I don’t go… I don’t go with my friends to parties. I spend more of my leisure time exercising [alone]. [FG3,M1]"

Men also reported less observance of diabetes self-care recommendations in social situations, such as eating fast food because family or friends typically enjoy it. One man talked about how he strays from self-management when with friends:

"I tend to go away from it [diabetes self-management] on the weekends…Everything…I have a beer here, there, and everywhere else, you know what I mean? I tend to go on a little bit. You know, when we get together. [FG3,M2]"

Both men and women were still readily able to accept their diabetes following an adjustment period after the initial diagnosis; both groups identified the importance of acceptance in obtaining knowledge about the disease and proper management in overcoming fears about their own health.

### Self-monitoring of blood glucose levels (SMBG)

Both men and women described their experiences practicing SMBG at home. They repeatedly discussed the importance of body cues in interpreting glucose levels – both hypoglycemic and hyperglycemic episodes. Men linked physical symptoms to fluctuations in blood glucose level. One noted,

I can feel…you know, the level of my blood…when my blood sugar is say, more than acceptable. [FG3,M1]. Furthermore, men and women associated variations in blood-glucose levels to other behaviors, particularly food selection. Participants acknowledged that certain foods caused their blood-glucose level to increase, and described using their blood glucose levels to gauge the impact of certain foods on glycemic control and modified subsequent eating behaviors as a result. Despite being able to link diet with blood glucose variations, both sexes found interpreting and reflecting upon factors that impact their blood sugar readings challenging and counter-intuitive at times. For example, one woman stated that she could,

"Eat half of a fruit, half of one fruit…and it [blood glucose] goes on up. I don’t know why. [FG1,F3]."

In another focus group, a male participant expressed his frustration with early morning blood glucose lows:

"I get up in the morning, 3…4.2. Why [does] it drop so much? I don’t understand why overnight it drops so much? [FG2,M4]."

We noted two important differences between males and females. Only men described experimenting with diet, exercise, medication, and insulin in response to blood glucose levels. One described how he reduced his required dosage of insulin by exercising:

"I would recommend exercise is maybe the number one part of the medication to diabetes. Let’s say that I exercise every evening, or let’s say 3 or 4 times [each week]. Like, my medication was cut almost a quarter. Because my sugar will drop all the way down to, even sometimes less than 5…but during the week, like some days when I can’t jog, then I have to really rely on the medication. [FG3,M1]"

Another experimented with diet:

"But we have to monitor ourselves and the food that we eat. That’s the way I do it. I eat twice a day…so I’ve learned from that, so I always eat from 12:00 in the afternoon, every day. I adjust myself. [FG1,M1]"

These findings suggest that self-adjustment of prescribed medication regimens based on modifications to diet and physical activity may be a common strategy that men use to self-manage diabetes.

Both sexes described worry, frustration and fear regarding SMBG but the nature of these discussions was qualitatively different. Women’s discussion about SMBG focused more on the affective components of SMBG including fears and anxieties associated with monitoring, the fear of needle pricks and the burden of regular testing. For example, one female reported,

"I am afraid of needles. But now I can do it. [FG5, F7]"

In contrast, men focused on the logistics and technical aspects of SMBG, including calibration of blood-glucose monitors, extracting and sizing a blood sample, and using test strips:

"Very often there is wide variation from [one of] my readings to the other. How do you calibrate these darn meters? Every time I buy strips, there is a test strip in there. Now, are you supposed to pick up a solution at the drug store? [FG2,M2]"

Another man discussed blood-sample size and learning with a trial-and-error process:

"One of the other things I found early in this experience is taking a proper sample. Like, when you prick yourself, you just get a little…but it has to be a full blood sample. Cause you get a low reading rate. And you say, ‘No no, this is not a good reading sample. Try the other finger.’ [FG2,M5]"

These findings demonstrate different aspects of SMBG that are of concern or interest to patients or that are a source of anxiety or fears.

### Diet struggles

Both male and female participants repeatedly voiced struggles with food restriction, moderation and integration of dietary recommendations, but dietary struggles were a much more prominent part of women’s self-care behavior. Women demonstrated significant nutrition knowledge gaps and expressed challenges with having to restrict favorite foods. One participant stated,

"It is very hard. It’s hard to stay off the sweets…because some people have diabetes, and they don’t like sweets. I love sweets! [FG4,F2]."

Another woman said,

"I know what to eat and what not to eat…I have to cut out starch…[It’s hard] that you can’t eat the things that you usually eat! [I,F9]."

Even when specifically told by their physicians that they could eat anything in moderation, women felt a need to abstain from certain foods in order to achieve optimal control:

"Yes, because the doctor told me I can eat everything, but in small portions. But I still don’t eat everything. [FG4,F1]."

A number of other female participants reported difficulties adjusting to appropriate food substitutes and the overall change to their diets: one participant noted that her major difficulty in managing her diabetes was adjusting to not eating as many desserts.

As a consequence of making these dietary changes, many women appear to progress through a period during which they mourn the loss of these foods from their diet. The feelings of loss and difficulty over abstaining from certain foods was especially prominent when participants discussed having to reduce or cut out foods from their specific cultural background:

"It’s a little difficult for me to break away from what I’m accustomed to eating…but it’s the passion [temptation] of trying to go back [to eating those foods]…it’s still there for me to go back. [FG1,F4]."

Moreover, women acknowledged their ‘dietary indiscretions’ (e.g. eating foods perceived as forbidden) as cheating on their diets. A female participant described one of the difficulties of having diabetes as,

"Not eating cookies or cakes…because I have a sweet tooth, but I cut back on that…once in a while, I do treat myself to a little bit. I cheat. But then I control how much. [I,F4]."

Women seemed to have developed a consensus with others in their focus groups when discussing the behavior of cheating. For example

"…and [I] find out [blood glucose levels are] going up, when I cheat a little, you know…When I see that and I stop cheating <other participants agreeing>…. [FG5,F1]"

To which another woman responded,

"Everybody cheats a bit, you know. [FG5,F4]."

The use of emotionally-charged language and description of dietary deviations as 'cheating' further supports the proposed complex relationship women have with food. Our results demonstrate that women with diabetes feel as though many foods are forbidden, which reveals the importance of emphasizing how to integrate foods into their diet and how to modify traditional cultural recipes so as to enable healthy and balanced approach to managing eating behaviors.

Men did not generally discuss food restriction, but rather discussed moderation and substitutions of foods perceived to be unhealthy. For example, one man discussed a substitution:

"Instead of eating white bread, you’re eating whole wheat or seven grain bread. [FG2,M3]"

Another reported,

"…because people love to eat sweets and everything, but I have to give it up. But the good thing about it is…we have Splenda…so if I need any…sweetener or compounds, we use Splenda at home for all the sweets and everything. [I,M8]."

Men also generally described deviating from their diets using less-self-deprecating language. For example,

"I tend to go away from it on the weekends. [FG3,M2]"

Another man said,

"I tend to splurge a little bit. [FG3,M3]"

Contrary to women, the men in this study appear to focus on moderation of foods and use more neutral language to describe nutrition self-care.

### Utilization of diabetes resources

"Men preferred more self-directed, independent education resources; for example,"

"I’ve read several books about this… [FG2,M2]"

And,

"Whenever I need some information, I go to all the Internet sites. The Internet… gives me very good material. So with that I manage myself. [I,M8]."

Even when men used more social information sources, they combined them with independent information seeking:

"I went to the Hospital…they gave me a booklet, which is very nice. One about what type of exercises you need to do and what type of diet management you need to do. That gave me a little bit of help…and at the same time, whenever I need some information, I go to all the sites, the Internet. [I,M8]"

In contrast, women tend to use more socially interactive education resources: the DEC, group classes, support groups (i.e., church exercise groups, foot clinics, weight loss programs) and their healthcare teams. For example, one woman discussed those resources that have been most helpful in helping her manage her diabetes:

"…Like talking to other people who have it…I’m in a senior’s group and then we have exercise. [I, F9]"

### Social support

Men and women both turn to their family and their physician for practical and moral support. They preferred their physicians to be aware of diabetes services and resources, to be willing to coordinate these resources for them and provide consistent follow-up including glycemic control, medication adjustments, and listening to patient’s personal stories. However, we noted differences among men and women in how participants discussed support from their families. Men repeatedly spoke in the plural about diet modifications, for example:

"What we did, from my own experience, is we changed the habits of all the family. [FG1,M5]"

Another man said,

"My wife is the controller in the house…She supports the whole thing, which I find very helpful. She says this is what you’re [going to] eat. And it doesn’t matter to me, I mean, because I eat anything she gives me. But she is in tune with the regime. This is what you should eat; this is what you shouldn’t eat. And you know, if you eat that, then you know what is going to happen. [FG2,M5]"

In contrast, most women did not discuss their spouse as a source of support and often felt the need to change their lifestyles without altering those of their families. One woman described how she balanced her own healthy diet with her family’s preferences, as she was the primary person in charge of food preparation for the family:

"I do the cooking, but I make sure when I cook it, it’s not going to affect any of us, and it’s still tasty whatever way I cook it. So nobody misses what I cook. [I, F4]"

Another woman described how isolating it was to eat foods different from the rest of her family and friends:

"So it’s really hard on family and friends with me, I feel like an alien and I can only eat certain types of food…so it’s very sad and difficult.” [FG5,F5]."

Unlike men who solely discussed their wives as support, women described mixed support from family members, especially other important women in their lives such as daughters, sisters and friends. One woman reported,

"My friends are pretty supportive…when we go out to dinner or something, they say, ‘maybe we can share a dessert’…and they remind me, ‘maybe you shouldn’t have that or whatever’. [I,F10]"

Other female participants emphasized predominantly nagging with regards to their diets from family. For example:

"No, my family is on top of me, don’t eat that, don’t eat that. If you go on, we will leave you… [FG5,F3]"

And,

"If they [family] see me, you know, eating anything that I know I shouldn’t eat, they’ll say, ‘Well you can’t have that,’ or something like that, you know. [I,F9]."

Regardless of positive or negative support, women did report influence from a wider group of people in their social support networks.

## Discussion

The focus of this study was to explore diabetes self-management experiences, specifically needs, challenges and barriers between men and women with T2DM. A salient finding was the difference between how men and women identify themselves as a person with diabetes or disclose their diagnosis to others. Women’s public life tends to run parallel to their private one; they disclose their diabetes more readily to others and overtly practice their nutrition self-care behaviors regardless of social context. Men, on the other hand, are much more private in their disclosure of diabetes and tend to be less observant of nutrition recommendations in social settings, where changes in diet patterns could potentially make their diagnosis public. According to Charmaz, men may strive to maintain their public, pre-diagnosis identity while adjusting their private identity to their illness [28]. Concealing their illness may help preserve traditional male values such as independence, autonomy, and ownership over decision-making. Liburd and colleagues examined the influence of perceived masculinity on diabetes self-management activities among African American men and suggested that a man’s desire to conform to the traditional masculine characteristics of autonomy, dominance, and stoicism may hinder self-care, glycemic control, and observance of treatment [29]. One’s private versus public identity may also influence their use of diabetes resources. For instance, men turn to books and the internet for further education over more socially interactive resources, such as counseling classes and support groups which are used more by women [30].

In contrast, women tend to disclose their diabetes more often to those in their social surroundings. Because traditional male characteristics do not apply to women, they may not view diabetes as negatively altering their identity. For example, the women in a study by Cagle et al. emphasized that they were not *diabetic* African American women, but rather, African American women *with diabetes [*[31]. This reflects an important attitude shift in chronic illness management in that individuals are separate from their diseases, not defined by them. Asbring examined the impact of chronic illness on gender identity among women with either chronic fatigue syndrome or fibromyalgia and found that women often experience early identity loss but later recognize the personal benefits to be gained from their condition, including greater insight into their lives [32]. Similarly, Koch et al. reported that many of their male participants believed diabetes changed them for the better because the diagnosis encouraged them to make positive lifestyle changes [33]. Although men may grapple with the threat of disclosing their diabetes to their public identity, they may also experience an identity trajectory of initial loss followed by a reframing process that involves separating the illness from the self. If men perceive diabetes as a threat to their masculine identity, healthcare providers may need to recognize this stigma and create an environment that initially addresses and works to dispel potential male stereotypes. In turn, men can be empowered to engage in social activities for the purpose of making healthier lifestyle choices and transition to making these lifestyle changes across varying social situations. Healthcare professionals can play a pivotal role in the transformation of self-identity following diagnosis by discouraging the labeling of people by diagnosis and introducing the illness as merely one aspect of their health that requires specific attention and management (‘individuals with diabetes’ versus ‘diabetics’).

The nature of familial support among participants in this study differed between men and women. Men identified their partners as their predominant source of social support and explained that their families contributed by adopting similar lifestyle changes, such as diet modifications. Liburd et al. also emphasized the role that wives play in men’s self-management of their diabetes, particularly regarding diet [29]. However, women described less spousal support but a wider network of support. Antonucci and Akiyama also found that among patients with diabetes women have larger support networks that include siblings and children while men rely predominantly on their spouses [34]. If men view their diabetes as a threat to their masculine identity, then their use of spousal and immediate family member support reduces the number of people aware of their disease and subsequently maintains their pre-diagnosis public identity. Women’s use of much larger social support networks aligns with their overt disclosure of disease and integration of diabetes into daily life. By utilizing a wide range of both formal and informal social supports, women may more easily make healthier lifestyle choices, regardless of social setting. Both men and women made use of social support available from immediate family members; however, women received encouragement or reminders of what not to eat or scrutiny over the amount eaten, whereas men’s household environment changed to support nutrition recommendations. Based on previous studies and our results, women appear to rely on a wider net of social support. As such, healthcare professionals should encourage the participation of family members, as well as other members of a person’s social network, in diabetes self-management education. In this way, educators help women build positive and well-informed support networks.

Based on the transcripts, it is clear that both men and women recognize the value of SMBG, link their physical symptoms to variations in glucose levels, and make appropriate behavioral adjustments. Men and women, however, vary in their concerns about SMBG; specifically, women focus on their fears and anxieties whereas men focus on the technical aspects of SMBG [35]. Our results support a similar finding that report men value the technical understanding of blood glucose control [33]. In spite of recommendations made by physicians and diabetes educators, men in our study were more proactive in experimenting with both SMBG and multiple aspects of diabetes self-management. The fact that both men, and to some degree women, experimented with self-care to some degree may indicate inquisitiveness and desire to learn from experience. Our results highlight the importance of not only adopting a patient-centered approach to diabetes care and counseling by addressing the salient issues that are identified by the patients themselves but also being knowledgeable and sensitive to particular gender interests such as calibration of glucose monitoring devices with men and fears and anxieties associated with SMBG among women. Healthcare professionals may find it helpful to share the common interests, fears and anxieties that men and women have with individuals who may be reluctant to ask questions or resistance to learning a new self-management skills such as SMBG or insulin administration.

Another important finding in our study was the notion among women that certain foods were forbidden rather than permitted in moderation, which might be the result of gender-specific attitudes held by women. Women in our study made repeated comments about missing or mourning certain foods; the perception that specific foods are forbidden may account for a mourning period that some women experience, during which time they adjust to complete abstinence from previously favorite or culturally preferred foods. Men, on the other hand, did not discuss mourning of foods; they appeared to focus on moderation of foods rather than restriction of any given food. However, men did discuss difficulties with adherence to dietary recommendations in social settings; these findings suggest that men too may also benefit from nutritional counseling with a focus on safe and effective strategies to integrate nutrition self-management skills during more challenging situations, or when away from home. These knowledge gaps might be the result of several factors including pre-conceived notions about food consumption, pre-occupation with restriction rather than moderation of foods perceived as unhealthy or conflicting information from multiple sources. According to research, women hold the misconception that nutrition therapy for weight loss requires over-restricting caloric intake and complete elimination of specific foods [36]. Therefore, if past dietary choices were guided by over-restriction, similar stringent eating patterns may continue after diagnosis. Dixey discusses women’s complex relationship with food, suggesting that although they are primarily responsible for the family’s food, they feel compelled to deny themselves certain foods in order to live up to the gender stereotype of slimness [36]. Polivy reports that food restriction can cause various cognitive disruptions, predisposing those who restrict intake to eat excessively - even binge - once restrictions are lifted; these behaviors can cause them to become overly preoccupied with and emotionally responsive to food [37]. Given the importance of nutrition self-care in diabetes self-management, healthcare providers should specifically assess individuals, in particularly women with diabetes, about food restriction, moderation and portion size. Based on our results, which demonstrate that women predominantly focus self-management on nutrition and dietary recommendations, guidance and education about nutrition self-management may be a more pertinent self-care strategy for women. Healthcare professionals may find that initiating counseling by addressing nutrition self-management interests may be an effective starting point for building rapport and trust with women. Empowering women to adopt a balanced approach to healthy eating might be more successfully obtained by providing counseling on inclusion of favorite foods and debunking food myths and misconceptions.

### Limitations

There are several important limitations that should be taken into account. The study population was pulled from a large, urban, culturally diverse city in Canada. As such, findings may not be representative of other and more culturally homogeneous populations of people with diabetes. Furthermore, we have limited information about the characteristics and time spent in the North American healthcare system of foreign-born participants and the amount of potential assimilation to western role expectations – this might act to potentially confound our results. We did not collect specific information regarding the ethnic and cultural background of those patients who reported being foreign-born, nor did we identify which cultural background participants identified with most strongly. Given the wide variations in cultural norms regarding diet, physical activity, and masculine and feminine gender roles and norms influencing health behaviors, the role of culture should be further investigated in future studies. The same interview guide was used for the focus groups and the individual interviews. There are potential differences that might occur due to this sampling strategy but using different methods can be potentially be a strength: for example, participants might mention more private self-care issues that might not come up in a focus group. While this mixed sampling methodology may be a limitation, we felt that it was more important to interview more participants than to include only those who were able to attend focus group. Finally, this study was based on secondary data and participants were recruited based on the degree of diabetes education participation at the DEC. For instance, approximately half of the participants had accessed the full range of diabetes education programming at the DEC, and others did not return for follow up after their initial visit. Therefore, our findings should be interpreted within the context of secondary data analysis.

## Conclusions

Our findings allowed us to develop a more comprehensive understanding of the self-management experiences, needs, challenges and barriers among men and women with diabetes. As the Canadian population ages, and diabetes affects an increasingly proportion of the population, patient empowerment and involvement in diabetes management will become even more crucial. This study will inform healthcare practice and counseling by illuminating the complex factors of gender differences. Our study found five overarching themes which represented differences in diabetes self-care among men and women: The five overarching themes identified were: increased self-identification and disclosure as a person living with diabetes among women, differences in attitudes towards and practical aspects of self-monitoring of blood glucose levels, increased struggles with diet and nutrition among women, utilization of more independent diabetes resources among men, and variations in included members among social support networks between men and women. As knowledge regarding differences among men and women and their management experience and needs increase gender-sensitive healthcare interventions can be better directed to improve self-management among women and men living with diabetes.

## **Appendix 1. Focus Group and Interview Topic Guide**

### **Note: Interview Guide was based upon the Brewer-Lowry Self-Management Framework**

Introduction

1. State your name, how long you’ve had diabetes for and why you decided to participate in the focus group.

Describe your overall experience living with and managing diabetes over the past year.

– What was going on in your life when you found out you had diabetes?

– Diet and nutrition

– Physical activity

Taking medication

– Coping with and managing stress

– Symptoms and complications

– Care received

– Watching blood pressure

2. Overall, how well do you feel and think you are able to manage your diabetes?

3. What were your difficulties with having diabetes over the past year?

– Diet: social gatherings, traveling, watching what you eat

– Personal life

– Treatment

– Future complications, life expectancy, morbidity, hyper/hypoglycemia

– Fears/anxieties about having diabetes over the past year (if not already brought up)

4. What kinds of support and resources have been most helpful to you in managing your diabetes?

– How do your family/friends help you?

– Family physician

– Books/magazines

5. Describe your experience during your visits to the Diabetes Education Center.

– Experience with the professionals and clerical staff

– Experience with the programs (one-on-one visits included)

6. How were your difficulties addressed during your visits to the Center?

– How were your fears/anxieties addressed during visits to the Center?

– Draw on prior answers for difficulties and fears

7. How would you improve the Diabetes Education Center (DEC) programs and services?

– Staff

– Resources

– Why didn’t you come back to the Center?

– What would keep you going back to the Center?

8. Overall, describe your experiences accessing healthcare in regards to your diabetes (aside from at the Diabetes Education Center).

– Physician

– Hospital

– Specialists

– RN, Registered Dietitian outside of DEC

9. Summarize the general themes of the focus group/interview and ask:

Conclusion and completion of demographic surveys, receipts, and distribution of honorarium compensation

– Is this a good representation of what was said?

– Does anyone else have anything to add?

– Does it spark any ideas for any of you?

## Abbreviations

DEC: Diabetes education centre; SMBG: Self-monitoring of blood glucose; T2DM: Type 2 diabetes mellitus.

## Competing interests

Ryerson University is providing the article-processing fees of this manuscript. The authors have no other competing interests to declare.

## Authors’ contributions

EG, MD and PB were involved in the conception and design of the study as well as acquisition of the data. RM, EG, MD and PB were involved in analysis and interpretation of the qualitative data. RM and EG made significant contributions to the statistical analyses, and drafting and revising of the manuscript during the writing process. RM, EG, MD and PB each provided valuable intellectual contributions to the manuscript during revisions. Finally, all authors have read and approved the final version of the manuscript for publication.

## Pre-publication history

The pre-publication history for this paper can be accessed here:

http://www.biomedcentral.com/1471-2296/13/122/prepub
